# Association of the cholesterol-HDL-glucose index with prevalent metabolic dysfunction-associated steatotic liver disease and its incremental metabolic-hepatic assessment value: a hospital-based cross-sectional study

**DOI:** 10.3389/fnut.2026.1862426

**Published:** 2026-07-01

**Authors:** Ziqian Song, Hao Sun, Cai Wang, Yuantao Qi, Yuan Liu

**Affiliations:** 1 Department of Epidemiology and Health Statistics, School of Public Health, Cheeloo College of Medicine, Shandong University, Jinan, China; 2 Qingdao Municipal Hospital, University of Health and Rehabilitation Sciences, Qingdao, China; 3 Binhai County People's Hospital, Yancheng, China; 4 Shandong Cancer Hospital and Institute, Shandong First Medical University and Shandong Academy of Medical Sciences, Jinan, China

**Keywords:** CHG, cholesterol-HDL-glucose index, health examination, MASLD, metabolic dysfunction-associated steatotic liver disease, opportunistic risk assessment

## Abstract

**Background:**

Metabolic dysfunction-associated steatotic liver disease (MASLD) is increasingly encountered in routine health examinations, where simple metabolic indices may help contextualize hepatic steatosis within broader cardiometabolic risk. The cholesterol-HDL-glucose (CHG) index integrates fasting glycemia and cholesterol-related information, but its association with prevalent MASLD and its incremental metabolic-hepatic assessment value in real-world health examination settings remain incompletely defined.

**Methods:**

We performed a cross-sectional analysis of an independent hospital-based analytic cohort comprising adults who underwent routine health examinations at the Health Examination Center of the Second Affiliated Hospital of Shandong First Medical University between 2024 and 2025. The final de-identified analytic dataset included 977 participants with complete questionnaire, anthropometric, laboratory, and abdominal ultrasonography data. The primary outcome was prevalent MASLD, operationally defined as ultrasonographic steatosis plus at least 1 cardiometabolic risk factor. We evaluated the association between CHG and prevalent MASLD using multivariable logistic regression, dose-response analyses, ROC comparisons with metabolic and steatosis-related indices, incremental model assessment, and sensitivity analyses.

**Results:**

The mean (SD) age was 44.13 (11.11) years, and 524 participants (53.6%) were men. MASLD was present in 343 participants (35.1%). Mean CHG was significantly higher in participants with MASLD than in those without MASLD [5.35 (0.22) vs 5.07 (0.25); *P* < 0.001]. In the primary multivariable model, each 1-SD increment in CHG was associated with higher odds of prevalent MASLD (OR, 2.23; 95% CI, 1.73–2.87; *P* < 0.001), and findings were consistent on the IQR scale and in sensitivity analyses. CHG discriminated prevalent MASLD better than fasting plasma glucose, total cholesterol, inverse HDL-C, and TyG; however, its performance was similar to TyG-WC and lower than FLI. Adding CHG to a baseline clinical model increased AUC from 0.84 to 0.85; continuous NRI and IDI improved, whereas categorical NRI was modest.

**Conclusions:**

Higher CHG was independently and dose-dependently associated with prevalent MASLD in adults undergoing hospital-based health examinations. CHG appears to capture a clinically relevant metabolic-hepatic signal, but the incremental gain beyond already strong clinical models is modest. Given the limited evidence supporting population-wide screening for steatosis alone, CHG should be interpreted as a low-cost adjunct for opportunistic metabolic-hepatic risk assessment rather than as a stand-alone diagnostic test or evidence sufficient for broad community MASLD screening. Prospective multicenter validation with longitudinal hepatic and cardiometabolic endpoints is warranted before CHG is adopted for risk-guided pathways.

## 1 Introduction

Metabolic dysfunction-associated steatotic liver disease (MASLD) has rapidly emerged as the preferred nomenclature for fatty liver disease associated with cardiometabolic dysfunction and has become one of the most common chronic liver disorders worldwide (1–5). Beyond hepatic steatosis, MASLD is increasingly recognized as a systemic metabolic disease linked to cardiometabolic morbidity, chronic kidney disease, malignancy, and premature mortality (6–13). Because the disease burden is substantial and often clinically silent in its early stages, efficient identification of high-risk individuals in non-specialist settings has become a major clinical and public health priority (4–13).

Routine health examination centers represent a particularly relevant setting for opportunistic MASLD detection because abdominal ultrasonography, standard biochemical testing, blood pressure assessment, and lifestyle questionnaires are commonly available during a single visit (10–18). However, although ultrasonography is practical and widely implemented, it remains operator-dependent and is less sensitive for mild steatosis, while more advanced imaging modalities are not always feasible for large-scale screening (15–18).

Because glycemic and lipid measurements are routinely obtained during health examinations, indices combining these variables may complement ultrasonographic findings and provide an efficient way to characterize metabolic-hepatic risk in routine practice.

The cholesterol-HDL-glucose (CHG) index is a recently proposed composite marker integrating total cholesterol (TC), high-density lipoprotein cholesterol (HDL-C), and fasting plasma glucose (FPG), thereby capturing complementary aspects of dysglycemia and cholesterol-related metabolic dysfunction (19–22). Emerging data suggest that CHG is associated with type 2 diabetes, broader cardiometabolic risk, and diabetic microvascular complications, and that it may provide a more integrated metabolic signal than isolated markers or some traditional composite indices (19–22). Given that hepatic steatosis develops within a network of insulin resistance, disordered lipid trafficking, and impaired lipoprotein metabolism, CHG is biologically plausible as a candidate marker for metabolic-hepatic assessment (6–9, 19–22).

Nevertheless, the current evidence base remains limited. The most directly relevant MASLD study to date evaluated CHG in two external Asian non-obese populations and demonstrated promising discriminatory performance, but additional validation in independent real-world hospital-based health examination populations remains necessary (19). The present study aimed to evaluate the association between CHG and prevalent MASLD, characterize dose-response patterns using both SD- and quartile-based models, compare CHG with conventional single markers and established or emerging steatosis-related indices, assess collinearity and incremental value cautiously, and explore whether CHG aligns more closely with steatosis/metabolic dysfunction than with FIB-4-defined fibrosis risk.

## 2 Materials and methods

### 2.1 Study design and participants

We conducted a cross-sectional analysis of a de-identified independent hospital-based analytic cohort consisting of adults who underwent routine health examinations at the Health Examination Center of the Second Affiliated Hospital of Shandong First Medical University between 2024 and 2025. The present analytic dataset represented one examination per participant and contained questionnaire, anthropometric, blood pressure, laboratory, and abdominal ultrasonography variables required for the prespecified analysis. After completeness review, 977 adults with complete data on all included variables were available for analysis.

**Figure 1:**
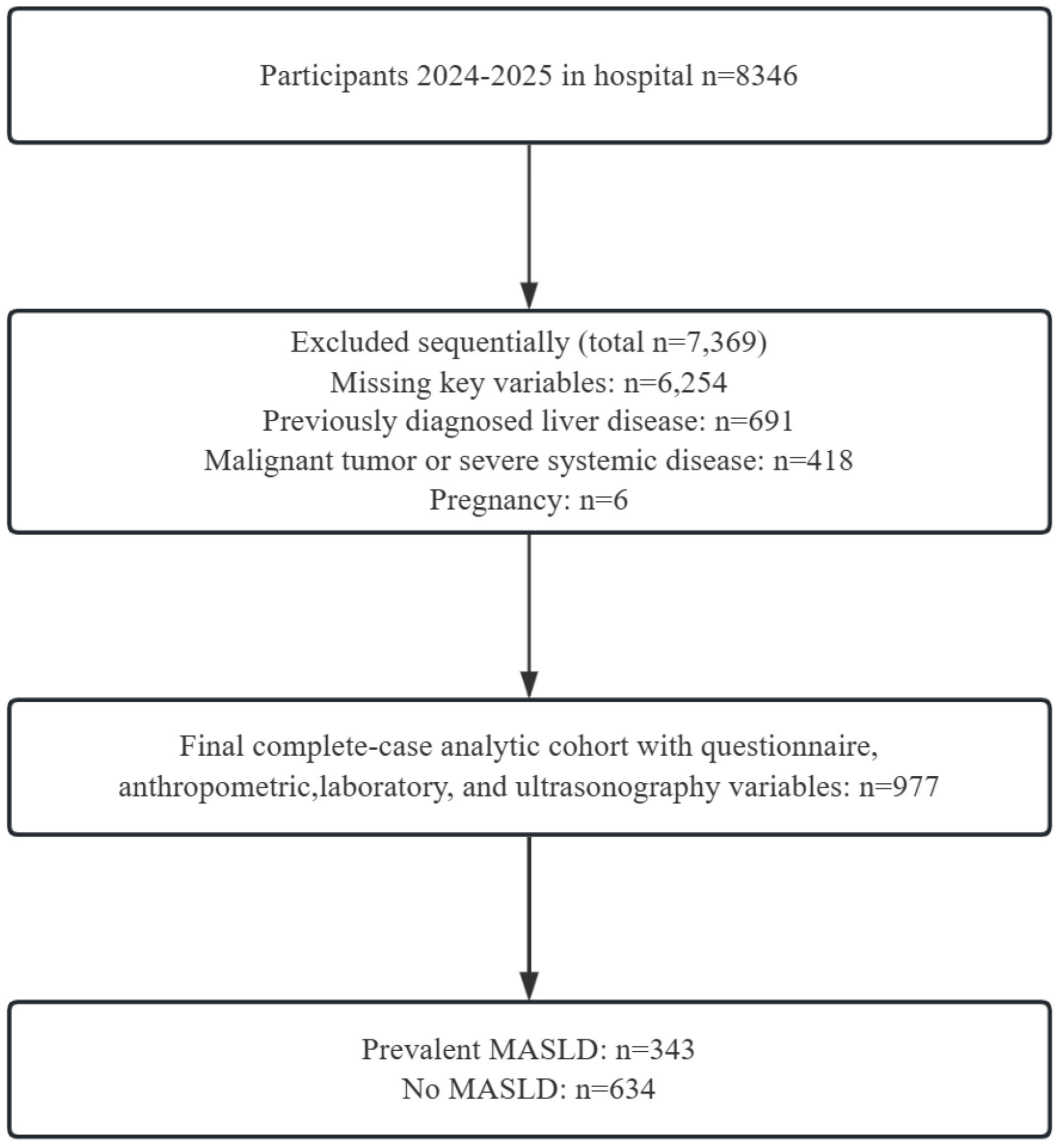
Participant flow diagram.

The participant selection process is summarized in Figure 1. A total of 8,346 routine health-examination participants were initially screened. We excluded 6,254 participants because of missing key variables, 691 participants because of previously diagnosed liver disease, 418 participants because of malignant tumor or severe systemic disease, and 6 participants because of pregnancy. After these exclusions, 977 adults with complete questionnaire, anthropometric, laboratory, and abdominal ultrasonography data were included in the final complete-case analytic cohort.

### 2.2 Clinical, laboratory, and ultrasonographic assessments

Standardized questionnaires were used to collect smoking status, drinking status, and regular exercise information. Anthropometric measurements included height, weight, body mass index (BMI), and waist circumference, and blood pressure was measured during the health examination visit using routine clinical procedures. Laboratory variables included fasting plasma glucose, glycated hemoglobin, TC, HDL-C, triglycerides, LDL-C, alanine aminotransferase, aspartate aminotransferase, gamma-glutamyl transferase, uric acid, creatinine, estimated glomerular filtration rate (eGFR), and platelet count. All biochemical measurements were obtained after overnight fasting as part of routine clinical testing.

Drinking status was collected through standardized questionnaire and patient self-report and was recorded as three categories: never drinking, occasional drinking, and frequent drinking. The de-identified analytic file did not retain quantitative alcohol intake in grams per day/week, beverage type, or drinking duration. Accordingly, although participants with previously diagnosed liver disease were excluded, the present analysis could not independently reconstruct ALD or MetALD alcohol-threshold exclusions from the analytic file; this limitation has been added explicitly.

Abdominal ultrasonography was used to ascertain hepatic steatosis. In keeping with conventional ultrasonographic criteria and contemporary health-check-based steatosis studies, hepatic steatosis was defined on the basis of standard echogenic findings (15–17). Steatosis severity was recorded in the analytic dataset as none, mild, moderate, or severe. After excluding participants with previously diagnosed liver disease, the present study operationalized prevalent MASLD as ultrasonographically detected steatosis plus at least 1 cardiometabolic risk factor in the final analytic dataset, consistent with contemporary MASLD nomenclature frameworks (1, 10–12, 19). Because quantitative alcohol information was unavailable, possible residual ALD/MetALD misclassification could not be completely excluded and is acknowledged as a limitation.

### 2.3 Operational definitions and derived indices

The prespecified cardiometabolic risk factors available in the analytic dataset were BMI at least 23 kg/m^2^, hyperglycemia (FPG ≥5.6 mmol/L or glycated hemoglobin ≥5.7%), elevated blood pressure (systolic blood pressure ≥130 mm Hg or diastolic blood pressure ≥85 mm Hg), hypertriglyceridemia (triglycerides ≥1.7 mmol/L), and low HDL-C (men < 1.0 mmol/L; women < 1.3 mmol/L), consistent with Asian and guideline-aligned operational definitions used in population-based steatotic liver disease research (1, 10–12, 14, 19). A secondary ultrasonographic steatosis outcome was defined irrespective of cardiometabolic criteria, and moderate-to-severe steatosis was defined as ultrasonographic grade 2 or 3.

The primary exposure was CHG, calculated as ln [(TC × FPG)/(2 × HDL-C)] after conversion of TC, FPG, and HDL-C from mmol/L to mg/dl, as previously described (19–21). For comparative purposes, TyG was calculated as ln [(fasting triglycerides × FPG)/2] in mg/dl (23, 24). We additionally calculated TyG-WC as TyG multiplied by waist circumference and TyG-WHtR as TyG multiplied by waist-to-height ratio to contextualize CHG against adiposity-augmented TyG derivatives (25). FLI was calculated using triglycerides, BMI, waist circumference, and GGT according to the original formula (26). HSI was calculated as 8 × ALT/AST + BMI + 2 if female + 2 if diabetes mellitus (27). Because no participant met diabetes-range FPG/HbA1c thresholds in the analytic dataset and medication/diagnosed diabetes variables were unavailable, the diabetes component of HSI was coded as 0; this HSI analysis should therefore be interpreted as an available-data approximation.

FIB-4 was calculated as age × aspartate aminotransferase / [platelet count × √alanine aminotransferase] (28). eGFR values in the analytic dataset were based on the 2021 CKD-EPI creatinine equation (29). Derived indices supplied in the analytic dataset were cross-validated against values reconstructed from raw variables and showed only trivial rounding differences.

### 2.4 Statistical analysis

Continuous variables are presented as mean (SD), and categorical variables are presented as number (percentage), in accordance with the prespecified reporting plan. Between-group comparisons were performed using Welch independent-samples *t* tests for continuous variables and χ^2^ tests for categorical variables. Because several variables deviated from normality by Shapiro-Wilk testing and/or visual inspection, we also performed Mann-Whitney U tests as sensitivity analyses for continuous variables. These checks did not materially change the baseline-characteristic conclusions.

We examined the association between CHG and prevalent MASLD using logistic regression with CHG modeled per 1-SD increment, per IQR increment, and by quartiles. Model 1 was unadjusted; Model 2 was adjusted for age and sex; and Model 3, the primary multivariable model, was adjusted for age, sex, smoking status, drinking status, regular exercise, BMI, systolic blood pressure, uric acid, and eGFR. ALT and GGT were not included in Model 3 because they may lie closer to hepatic injury or inflammatory activity and could overadjust the hepatic phenotype. An additional liver-enzyme-adjusted model included ALT and GGT to evaluate robustness under a more fully adjusted specification.

To assess dose-response relationships, restricted cubic splines with knots located at the 5th, 35th, 65th, and 95th percentiles of the CHG distribution were fitted using Model 3 covariates. Variance inflation factors (VIFs) were calculated for the liver-enzyme-adjusted model including CHG to assess multicollinearity. Discriminatory performance was quantified using ROC analysis and AUC. CHG was compared with FPG, TC, inverse HDL-C, TyG, TyG-WC, TyG-WHtR, FLI, and HSI, and differences in AUC were assessed using the DeLong method (30).

We evaluated whether adding CHG to a baseline clinical model improved performance using AUC, continuous NRI, IDI, and an exploratory categorical NRI. Because no universally accepted predicted-risk categories exist for cross-sectional MASLD detection, categorical NRI was calculated using pragmatic low/intermediate/high predicted-risk categories of < 20%, 20–40%, and ≥40%, and was interpreted cautiously. Decision curve analysis and calibration plots were considered supplementary and were interpreted descriptively rather than as evidence of definitive clinical decision improvement.

Prespecified subgroup analyses were performed by sex, age (< 50 vs ≥50 years), BMI (< 24 vs ≥24 kg/m^2^), and regular exercise status, with multiplicative interaction terms used to test heterogeneity. Sensitivity analyses redefined the outcome as ultrasonographic steatosis irrespective of cardiometabolic criteria, excluded overlap-only MASLD cases, replaced BMI with waist circumference, used robust Poisson regression to estimate prevalence ratios, restricted the outcome to moderate-to-severe steatosis, and explored steatosis plus raised ALT. Raised ALT was defined using sex-specific thresholds (>30 U/L for men and >19 U/L for women) for epidemiological sensitivity analysis; this outcome should not be interpreted as histologic MASH. FIB-4 alignment was assessed using Spearman correlation, FIB-4 category distributions (< 1.30, 1.30–2.67, >2.67), and a logistic model for FIB-4 ≥1.30.

All tests were 2-sided, and *P* < 0.05 was considered statistically significant. Analyses were performed in Python 3.13 using pandas, SciPy, statsmodels, scikit-learn, and matplotlib.

## 3 Results

### 3.1 Participant characteristics

The final analytic cohort included 977 adults, of whom 524 were men. The mean (SD) age was 44.13 years. Prevalent MASLD was identified in 343 participants, whereas ultrasonographic steatosis of any grade was present in 362. Steatosis grades were distributed as mild in 75 participants, moderate in 157, and severe in 130. Table 1 summarizes baseline characteristics according to MASLD status and includes test statistics and nonparametric sensitivity *P* values.

**Table 1:** Baseline characteristics according to prevalent MASLD status.

Characteristic	Overall (*N* = 977)	No MASLD (*n* = 634)	MASLD (*n* = 343)	Test statistic	*P* value	Mann–Whitney *P*
Age, years	44.13 (11.11)	42.33 (10.83)	47.47 (10.84)	−7.08	< 0.001	< 0.001
Height, cm	165.46 (8.01)	164.65 (7.99)	166.95 (7.83)	−4.35	< 0.001	< 0.001
Weight, kg	67.67 (11.47)	63.90 (10.25)	74.64 (10.28)	−15.59	< 0.001	< 0.001
BMI, kg/m^2^	24.62 (3.09)	23.50 (2.81)	26.69 (2.44)	−18.52	< 0.001	< 0.001
Waist circumference, cm	83.08 (7.44)	80.75 (6.97)	87.40 (6.27)	−15.21	< 0.001	< 0.001
SBP, mmHg	120.71 (12.29)	118.55 (12.15)	124.69 (11.54)	−7.80	< 0.001	< 0.001
DBP, mmHg	74.64 (8.97)	73.39 (8.76)	76.95 (8.92)	−6.00	< 0.001	< 0.001
FPG, mmol/L	5.22 (0.47)	5.08 (0.44)	5.46 (0.43)	−13.06	< 0.001	< 0.001
HbA1c, %	5.47 (0.22)	5.44 (0.21)	5.53 (0.22)	−5.66	< 0.001	< 0.001
TC, mmol/L	5.11 (0.61)	4.96 (0.57)	5.39 (0.57)	−11.26	< 0.001	< 0.001
HDL-C, mmol/L	1.36 (0.21)	1.42 (0.20)	1.25 (0.18)	12.95	< 0.001	< 0.001
TG, mmol/L	1.37 (0.70)	1.20 (0.60)	1.68 (0.77)	−10.04	< 0.001	< 0.001
LDL-C, mmol/L	3.22 (0.67)	3.09 (0.64)	3.47 (0.65)	−8.93	< 0.001	< 0.001
ALT, U/L	24.70 (13.30)	19.77 (9.13)	33.81 (14.91)	−15.91	< 0.001	< 0.001
AST, U/L	21.66 (7.25)	19.82 (6.13)	25.07 (7.91)	−10.68	< 0.001	< 0.001
GGT, U/L	31.12 (22.17)	25.03 (14.43)	42.38 (28.67)	−10.51	< 0.001	< 0.001
Uric acid, μmol/L	330.78 (67.71)	311.01 (62.73)	367.31 (61.15)	−13.61	< 0.001	< 0.001
Creatinine, μmol/L	73.52 (14.30)	70.67 (13.80)	78.81 (13.71)	−8.84	< 0.001	< 0.001
eGFR, mL/min/1.73 m^2^	102.88 (13.30)	105.62 (12.74)	97.81 (12.84)	9.10	< 0.001	< 0.001
Platelets, × 10?/L	236.17 (36.63)	239.93 (35.58)	229.23 (37.57)	4.33	< 0.001	< 0.001
CHG index	5.17 (0.28)	5.07 (0.25)	5.35 (0.22)	−17.89	< 0.001	< 0.001
TyG index	8.53 (0.52)	8.38 (0.50)	8.80 (0.45)	−13.55	< 0.001	< 0.001
FIB-4	0.94 (0.55)	0.90 (0.52)	1.02 (0.58)	−3.29	0.001	< 0.001
Sex				39.13	< 0.001	
Female	453 (46.37)	341 (53.79)	112 (32.65)			
Male	524 (53.63)	293 (46.21)	231 (67.35)			
Smoking status				14.42	< 0.001	
Never	717 (73.39)	488 (76.97)	229 (66.76)			
Former	38 (3.89)	17 (2.68)	21 (6.12)			
Current	222 (22.72)	129 (20.35)	93 (27.11)			
Drinking status				7.23	0.027	
Never	603 (61.72)	401 (63.25)	202 (58.89)			
Occasional	139 (14.23)	97 (15.30)	42 (12.24)			
Frequent	235 (24.05)	136 (21.45)	99 (28.86)			
Regular exercise			2	3.77	0.052	
No	579 (59.26)	361 (56.94)	218 (63.56)			
Yes	398 (40.74)	273 (43.06)	125 (36.44)			
BMI ≥23 kg/m^2^				=153.88	< 0.001	
No	302 (30.91)	282 (44.48)	20 (5.83)			
Yes	675 (69.09)	352 (55.52)	323 (94.17)			
Hyperglycemia				71.08	< 0.001	
No	684 (70.01)	502 (79.18)	182 (53.06)			
Yes	293 (29.99)	132 (20.82)	161 (46.94)			
Elevated blood pressure				52.48	< 0.001	
No	661 (67.66)	480 (75.71)	181 (52.77)			
Yes	316 (32.34)	154 (24.29)	162 (47.23)			
Hypertriglyceridemia				64.66	< 0.001	
No	745 (76.25)	535 (84.38)	210 (61.22)			
Yes	232 (23.75)	99 (15.62)	133 (38.78)			
Low HDL–C				12.93	< 0.001	
No	843 (86.28)	566 (89.27)	277 (80.76)			
Yes	134 (13.72)	68 (10.73)	66 (19.24)			

Data are presented as mean (SD) or No. (%). Test statistics are Welch t values for continuous variables and χ^2^ values for categorical variables. Mann-Whitney P values are shown as nonparametric sensitivity checks for continuous variables.

Compared with participants without MASLD, those with MASLD were older and more frequently male and had higher adiposity indices, blood pressure, glycemic parameters, atherogenic lipid measures, liver enzymes, uric acid, creatinine, CHG, TyG, and FIB-4, together with lower HDL-C and lower eGFR. Mann-Whitney U sensitivity analyses yielded the same qualitative conclusions for all continuous baseline variables.

### 3.2 Association between CHG and prevalent MASLD

In unadjusted analyses, each 1-SD increase in CHG was associated with nearly 4-fold higher odds of prevalent MASLD (OR, 3.97; 95% CI, 3.24–4.87; *P* < 0.001). The association remained strong after adjustment for age and sex (OR, 3.62; 95% CI, 2.91–4.50; *P* < 0.001). In the primary multivariable model, each 1-SD increment in CHG remained independently associated with prevalent MASLD (OR, 2.23; 95% CI, 1.73–2.87; *P* < 0.001). In the additional liver-enzyme-adjusted model, the association was similar (OR, 2.28; 95% CI, 1.73–3.01; *P* < 0.001).

On the IQR scale, each IQR increment in CHG was associated with higher odds of prevalent MASLD (OR, 2.80; 95% CI, 2.02–3.88; *P* < 0.001) (Table 2). Quartile-based analyses showed a graded pattern, and the trend per quartile was statistically significant (Supplementary Table S1). The trend estimate provides an interpretable summary of the ordinal dose-response pattern, while the upper-quartile category-specific ORs should be read with attention to their wider confidence intervals.

**Table 2:** Association between the CHG index and prevalent MASLD using continuous exposure scales.

Dependent variable	Independent variable	Model 1		Model 2		Model 3	
		OR (95% CI)	*P*–value	OR (95% CI)	*P*–value	OR (95% CI)	*P*–value
MASLD	Per 1–SD increase	3.97 (3.24–4.87)	< 0.001	3.62 (2.91–4.50)	< 0.001	2.23 (1.73–2.87)	< 0.001
	Per IQR increase	5.91 (4.55–7.67)	< 0.001	5.24 (3.96–6.93)	< 0.001	2.80 (2.02–3.88)	< 0.001

Model 1 was unadjusted. Model 2 was adjusted for age and sex. Model 3 was adjusted for age, sex, smoking status, drinking status, regular exercise, BMI, systolic blood pressure, uric acid, and estimated glomerular filtration rate. Quartile-based estimates and trend-per-quartile results are presented in Supplementary Table S1.

**Figure 2:**
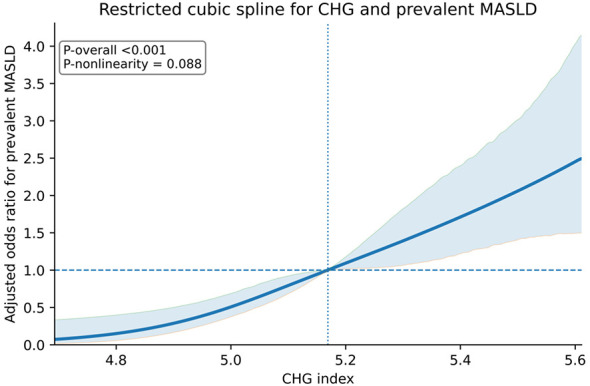
**R**estricted cubic spline depicting the association between CHG and prevalent MASLD in Model 3. The display is restricted to the 5th-95th percentile range of CHG to reduce sparse-tail instability of the confidence band.

Restricted cubic spline analysis using Model 3 demonstrated a significant overall association between CHG and prevalent MASLD (*P* overall < 0.001) without strong evidence of nonlinearity (*P* nonlinear = 0.088) (Figure 2).

### 3.3 Discriminatory performance and incremental assessment value

**Figure 3:**
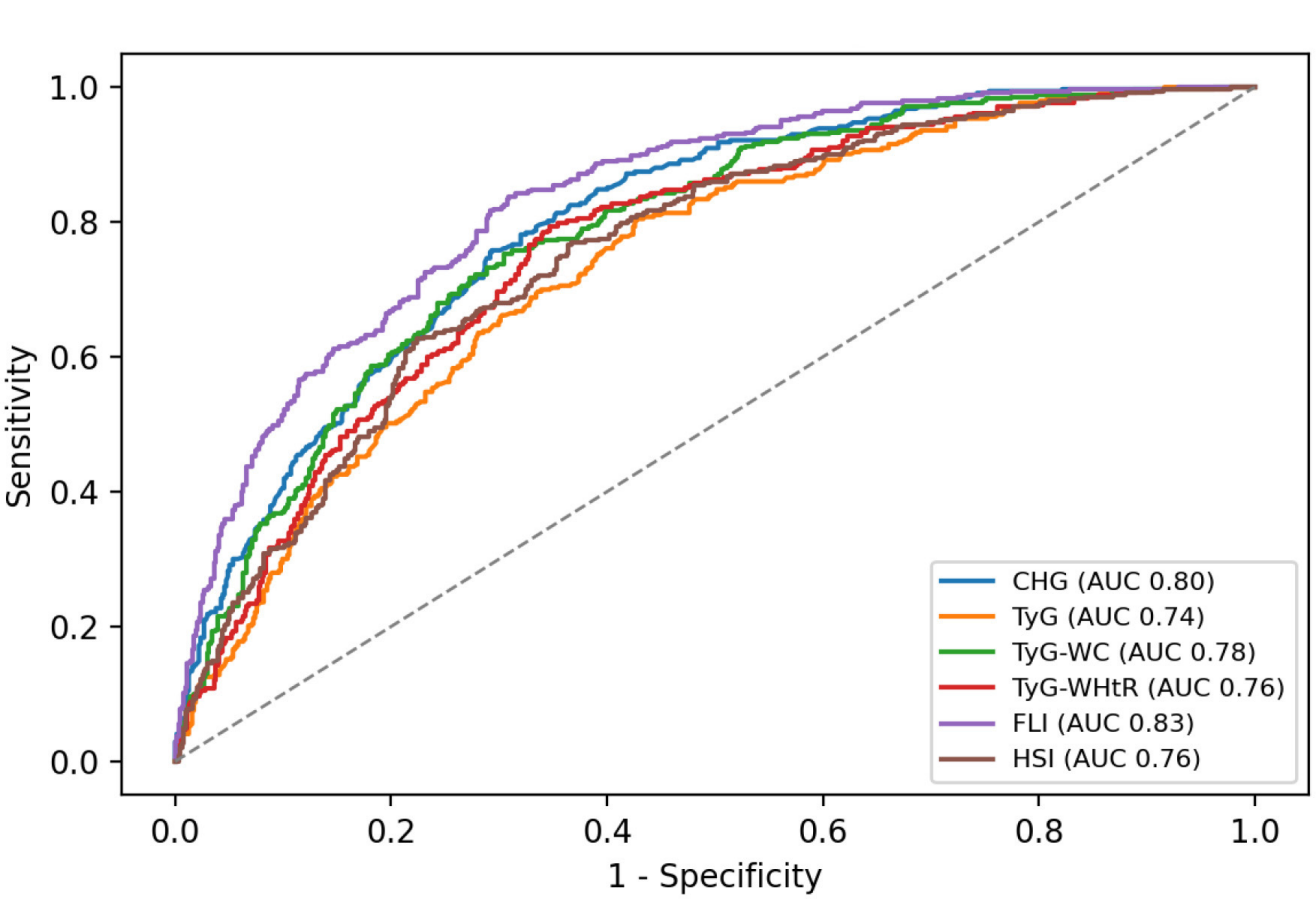
ROC curves for CHG and selected comparator indices.

In ROC analysis, CHG showed good discrimination for prevalent MASLD and outperformed the evaluated single markers and TyG. However, CHG was not universally superior to metabolic or steatosis-related indices: its AUC was not significantly different from TyG-WC, was only modestly higher than TyG-WHtR, and was lower than FLI. These results place CHG in a more cautious context as a metabolic-hepatic indicator rather than a replacement for established steatosis indices. The complete AUC comparison is presented in Table 3, and the corresponding ROC curves are shown in Figure 3.

**Table 3:** Discriminatory performance of CHG and comparator markers/indices for prevalent MASLD.

Marker/index	AUC (95% CI)	*P*–value vs CHG
CHG	0.80 (0.77–0.83)	Reference
FPG	0.73 (0.70–0.76)	< 0.001
TC	0.70 (0.66–0.73)	< 0.001
1/HDL–C	0.72 (0.69–0.76)	< 0.001
TyG	0.74 (0.71–0.77)	< 0.001
TyG–WC	0.78 (0.75–0.81)	0.307
TyG–WHtR	0.76 (0.73–0.79)	0.022
FLI	0.83 (0.81–0.86)	0.011
HSI	0.76 (0.72–0.79)	0.028

In the primary baseline clinical model, AUC increased from 0.84 (95% CI, 0.81–0.86) to 0.85 (95% CI, 0.83–0.88) after adding CHG (*P* = 0.001). Continuous NRI was 0.39 (95% CI, 0.27–0.51), and IDI was 0.04 (95% CI, 0.02–0.05). Categorical NRI using < 20%, 20–40%, and ≥40% risk categories was modest (0.07; 95% CI, 0.02–0.13). These incremental-value results are summarized in Table 4, and the ROC comparison between the primary clinical model and the CHG-augmented model is provided in Supplementary Figure S1.

**Table 4:** Incremental value of adding CHG to clinical models.

Model performance^*^	Estimate (95% CI)	*P*–value/interpretation
Primary clinical model (no ALT/GGT)	0.84 (0.81–0.86)	Reference
Primary clinical model + CHG	0.85 (0.83–0.88)	< 0.001
Continuous NRI	0.39 (0.27–0.51)	< 0.001^*^
Categorical NRI (< 20%, 20–40%, ≥40%)	0.07 (0.02–0.13)	Exploratory
IDI	0.04 (0.02–0.05)	< 0.001^*^
Exploratory liver–enzyme–adjusted model	0.89 (0.87–0.91)	Reference
Exploratory liver–enzyme–adjusted model + CHG	0.90 (0.88–0.92)	0.003
Exploratory categorical NRI in liver–enzyme model	0.04 (-0.01–0.09)	Exploratory

Bootstrap intervals excluding 0 are reported as statistically supportive; categorical NRI is exploratory because no accepted cross-sectional MASLD risk categories exist.

Under the additional liver-enzyme-adjusted specification, the AUC increased from 0.89 to 0.90 (*P* = 0.003), with continuous NRI 0.35 and IDI 0.03; however, categorical NRI was smaller (0.04) and its bootstrap 95% CI crossed 0. These findings support a statistically detectable but clinically modest incremental gain when the baseline model is already strong. The decision-curve assessment of the primary model and the CHG-augmented model is shown in Supplementary Figure S2, and the corresponding calibration assessment is shown in Supplementary Figure S3.

### 3.4 Collinearity, FIB-4 alignment, subgroup analyses, and sensitivity analyses

VIF analysis did not suggest problematic multicollinearity. In the exploratory liver-enzyme-adjusted model including CHG, the maximum VIF was 3.46 (uric acid), and the VIF for CHG was 2.07. The detailed VIF results are provided in Supplementary Table S2.

FIB-4 alignment analyses suggested that CHG primarily reflects metabolic/steatotic burden rather than fibrosis-stage risk. CHG showed only a modest correlation with FIB-4 (Spearman *r* = 0.28; *P* < 0.001). FIB-4 categories were distributed as < 1.30 in 767 participants, 1.30–2.67 in 201 participants, and >2.67 in 9 participants. Although CHG increased across FIB-4 categories, the adjusted association with FIB-4 ≥1.30 was not statistically significant after clinical covariate adjustment (OR, 1.22; 95% CI, 0.92–1.62; *P* = 0.168). The distribution of CHG and MASLD across FIB-4 categories is presented in Supplementary Table S3.

**Figure 4:**
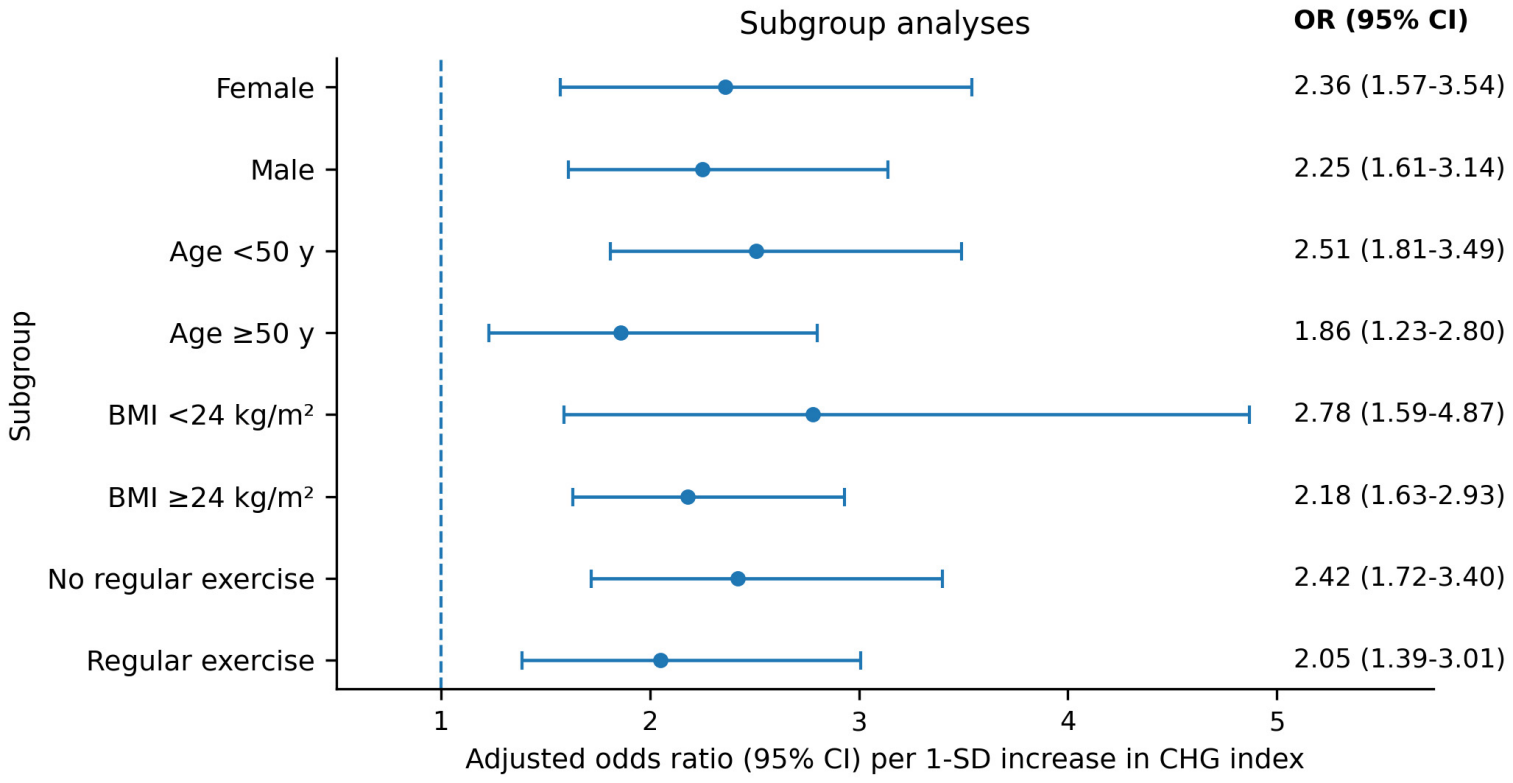
Subgroup analyses of the association between CHG and prevalent MASLD.

In subgroup analyses, the association between CHG and prevalent MASLD was directionally consistent across sex, age, BMI, and regular exercise strata, with no statistically significant interaction. Because sparse cells produced separation in some female smoking strata, subgroup findings should be interpreted as supportive rather than primary evidence. The subgroup estimates are displayed in Figure 4.

Sensitivity analyses yielded materially consistent associations across alternative outcome definitions and modeling strategies, as summarized in Table 5.

**Table 5:** Sensitivity analyses using Model 3 unless otherwise specified.

Sensitivity analysis	Effect estimate (95% CI)	*P*–value
Outcome redefined as ultrasonographic steatosis	OR 2.14 (1.68–2.72)	< 0.001
Excluding overlap–only MASLD cases (*n* = 9)	OR 2.12 (1.64–2.75)	< 0.001
Replacing BMI with waist circumference	OR 2.65 (2.08–3.37)	< 0.001
Continuous CHG model re–estimated using robust Poisson regression	PR 1.36 (1.22–1.51)	< 0.001
Outcome redefined as moderate–to–severe steatosis	OR 2.60 (1.97–3.44)	< 0.001
Outcome redefined as steatosis plus raised ALT	OR 1.67 (1.30–2.15)	< 0.001

All sensitivity analyses used the primary covariate framework unless otherwise specified. The steatosis plus raised ALT analysis used sex-specific ALT thresholds and was exploratory; it should not be interpreted as histologically confirmed MASH.

## 4 Discussion

In this hospital-based cross-sectional study of adults undergoing routine health examinations, higher CHG was independently and dose-dependently associated with prevalent MASLD. The association remained robust after multivariable adjustment and was observed under multiple analytic specifications. However, our interpretation is cautious. Because current evidence does not support population-wide screening for steatosis alone in all adults, and because long-term outcomes are driven more strongly by steatohepatitis, fibrosis, and systemic cardiometabolic risk than by ultrasound-defined steatosis alone, CHG should be viewed as a low-cost metabolic-health indicator with hepatic outcome relevance in opportunistic health examination settings, not as a stand-alone diagnostic test or proof of clinically meaningful improvement in broad MASLD screening.

Our findings extend the emerging CHG literature in several ways. Prior work has linked CHG to prevalent diabetes, cardiometabolic risk, and diabetic microvascular complications (19–22), and a recent study reported promising performance of CHG for identifying MASLD in external Asian non-obese populations (19). The present study provides complementary real-world evidence from an independent hospital-based analytic cohort derived from routine health examinations in China, encompassing a broader adult BMI distribution and incorporating questionnaire-derived lifestyle variables, comparator-marker evaluation, collinearity assessment, quartile-based modeling, and bias-oriented sensitivity analyses.

The biological plausibility of CHG as a MASLD-related metric is strong. Hepatic steatosis develops at the intersection of insulin resistance, increased hepatic glucose flux, *de novo* lipogenesis, impaired very-low-density lipoprotein handling, and broader disturbances in cholesterol and lipoprotein metabolism (6–10, 31–34). Elevated fasting glucose captures one dimension of impaired insulin sensitivity and hepatic metabolic inflexibility, whereas lower HDL-C may reflect reduced reverse cholesterol transport and a more atherogenic lipoprotein milieu. Total cholesterol adds further information on circulating cholesterol burden and metabolic dysregulation. A composite index that integrates these domains may therefore capture clinically meaningful metabolic stress more effectively than any single component alone.

The expanded comparator analyses are important for interpreting the practical role of CHG. CHG outperformed FPG, TC, inverse HDL-C, and TyG, but it did not outperform all modern or established steatosis-related indices. Its AUC was not significantly better than TyG-WC, and although it was statistically higher than TyG-WHtR in this dataset, the absolute difference was small. FLI had a higher AUC than CHG, likely because FLI directly incorporates BMI, waist circumference, triglycerides, and GGT, variables closely aligned with ultrasound-defined steatosis. These findings suggest that CHG may be convenient when TC, HDL-C, and FPG are readily available, but it should not be regarded as an optimal or universal marker for overall metabolic health assessment, nor as a replacement for steatosis-focused indices such as FLI.

The incremental value analyses should also be interpreted cautiously. Adding CHG to a baseline clinical model yielded statistically significant improvements in AUC, continuous NRI, and IDI. However, the absolute AUC gain was small, particularly when liver enzymes were included, and the categorical NRI was modest. These results do not establish that adding CHG would materially change clinical decisions in settings where basic clinical, anthropometric, and liver-enzyme information is already available. Instead, CHG may be most useful as an inexpensive adjunct for metabolic-hepatic risk stratification in opportunistic health examination workflows, especially where calculation can be automated from routine fasting biochemistry.

Collinearity assessment provides context for interpreting the multivariable findings. VIF values were below conventional thresholds, and CHG itself had a VIF of approximately 2. Nevertheless, the term “independent” should not be interpreted as meaning that CHG is biologically separate from the core metabolic syndrome pathway. CHG is intentionally constructed from glucose and lipid components and therefore represents an integrated metabolic signal rather than a mechanistically distinct disease axis.

The FIB-4 analyses further clarify what CHG is likely capturing. CHG correlated only modestly with FIB-4 and was not independently associated with FIB-4 ≥1.30 after clinical covariate adjustment, whereas it remained associated with prevalent MASLD and with steatosis plus raised ALT in sensitivity analyses. These findings suggest that CHG is more closely aligned with metabolic steatosis and hepatic biochemical activity than with fibrosis-stage classification. Prospective studies with elastography, MRI-PDFF, biopsy, or longitudinal liver-related outcomes are needed to determine whether CHG has prognostic value beyond steatosis detection.

This study has several strengths. First, it was based on an independent hospital-based health examination analytic cohort with complete data on questionnaire variables, anthropometric measures, biochemical markers, and ultrasonography. Second, we used a clinically pragmatic outcome framework anchored in routine health examination practice. Third, the analysis moved beyond simple association testing to incorporate dose-response modeling, quartile-based estimates with trend assessment, expanded comparator-marker evaluation, incremental model assessment, VIF assessment, FIB-4 alignment, and multiple sensitivity analyses. Fourth, the primary exposure is inexpensive and easily reproducible from tests already performed in routine practice.

Several limitations should be considered. The cross-sectional design precludes causal inference and does not address incident MASLD, longitudinal fibrosis progression, cardiovascular events, or liver-related outcomes. The study was conducted in a single hospital-based health examination setting, which may limit generalizability to community-based primary care screening or other populations. The population attending health examinations is inherently subject to selection bias; therefore, the findings are most applicable to opportunistic assessment within similar health-check workflows and should not be generalized to population-wide MASLD screening without external validation. MASLD was operationalized using abdominal ultrasonography rather than liver biopsy, MRI-PDFF, or elastography. Although participant-flow information was available and participants with previously diagnosed liver disease were excluded, the de-identified analytic dataset did not contain liver-disease subtype information, medication exposure variables, diagnosed diabetes status, viral hepatitis serology, or quantitative alcohol intake; therefore, residual disease misclassification, including possible ALD/MetALD misclassification, and residual confounding cannot be excluded. Finally, although component-overlap bias was addressed through sensitivity analyses, partial definitional coupling remains possible because CHG includes glucose and HDL-C information that overlaps with cardiometabolic MASLD criteria.

## 5 Conclusion

Higher CHG was independently associated with prevalent MASLD in adults undergoing hospital-based routine health examinations, and the association was dose-dependent and robust across multiple analytic scenarios. CHG appears to capture an integrated metabolic-hepatic signal and may provide low-cost adjunctive information in opportunistic health examination settings. However, its incremental value beyond strong clinical models is modest, it does not outperform all established steatosis or anthropometry-augmented metabolic indices, and it should not be promoted as a stand-alone MASLD screening tool. Prospective multicenter validation with longitudinal hepatic and cardiometabolic endpoints is warranted.

## Data Availability

The original contributions presented in the study are included in the article/Supplementary material, further inquiries can be directed to the corresponding author.
