# Supplementary material for: Association of the cholesterol-HDL-glucose index with prevalent metabolic dysfunction-associated steatotic liver disease and its incremental metabolic-hepatic assessment value: a hospital-based cross-sectional study
Source: Front Nutr. 2026 Jul 1;13:1862426. doi: 10.3389/fnut.2026.1862426 (PMC13368752; doi:10.3389/fnut.2026.1862426)

| **CHG Quartile** | **Model 1** | | **Model 2** | | **Model 3** | |
| --- | --- | --- | --- | --- | --- | --- |
|  | **OR (95% CI)** | **P-value** | **OR (95% CI)** | **P-value** | **OR (95% CI)** | **P-value** |
| **Q1** | Reference |  | Reference |  | Reference |  |
| **Q2** | 3.74 (2.09-6.67) | <0.001 | 3.44 (1.92-6.16) | <0.001 | 2.16 (1.17-3.98) | 0.014 |
| **Q3** | 11.33 (6.51-19.70) | <0.001 | 9.19 (5.21-16.20) | <0.001 | 3.76 (2.03-6.95) | <0.001 |
| **Q4** | 26.61 (15.21-46.58) | <0.001 | 20.02 (11.16-35.90) | <0.001 | 5.64 (2.93-10.86) | <0.001 |
| **Trend per quartile** | 2.87 (2.47-3.35) | <0.001 | 2.60 (2.21-3.07) | <0.001 | 1.72 (1.42-2.09) | <0.001 |

**Supplementary Table S1.** Quartile-based association between the CHG index and prevalent MASLD

*Q1 was the reference category. Quartile analyses showed a graded association, and the trend per quartile provides an interpretable summary of the ordinal dose-response pattern. Category-specific ORs and 95% CIs, especially in upper quartiles, were comparatively large and wide, indicating limited precision; these estimates should therefore be interpreted alongside the continuous and trend analyses rather than as the sole primary effect estimates.*

**Supplementary Table S2.** Variance inflation factors for the exploratory liver-enzyme-adjusted model including CHG

| **Variable** | **VIF** |
| --- | --- |
| Uric acid | 3.46 |
| Sex | 2.98 |
| BMI | 2.45 |
| Age | 2.31 |
| EGFR | 2.19 |
| CHG | 2.07 |
| ALT | 1.40 |
| Smoke | 1.33 |

**Supplementary Table S3.** Distribution of CHG and MASLD across FIB-4 categories

| **FIB-4 category** | **No. of participants** | **MASLD cases** | **CHG mean (SD)** |
| --- | --- | --- | --- |
| <1.30 | 767 | 257 | 5.14 (0.27) |
| 1.30-2.67 | 201 | 80 | 5.29 (0.26) |
| >2.67 | 9 | 6 | 5.44 (0.33) |

**Supplementary Figures**

Supplementary Figures S1-S3 provide additional model-performance displays, including ROC curves for the primary clinical model and CHG-augmented model, decision curve analysis, and calibration plots.

**Supplementary Figure S1.** ROC curves comparing the primary clinical model with the primary clinical model plus CHG for identifying prevalent MASLD.


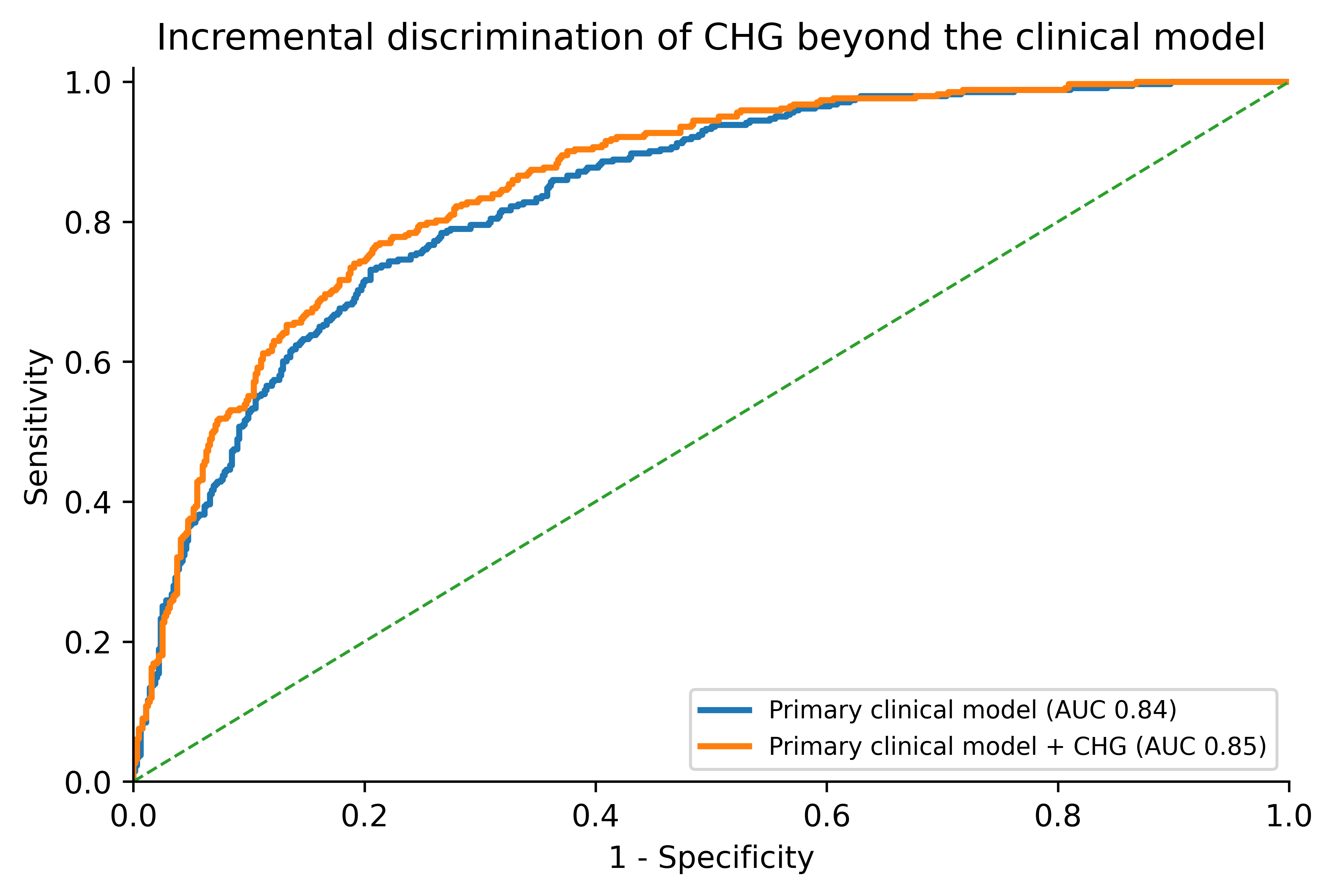


**Supplementary Figure S2.** Decision curve analysis for the primary clinical model and the primary clinical model plus CHG.


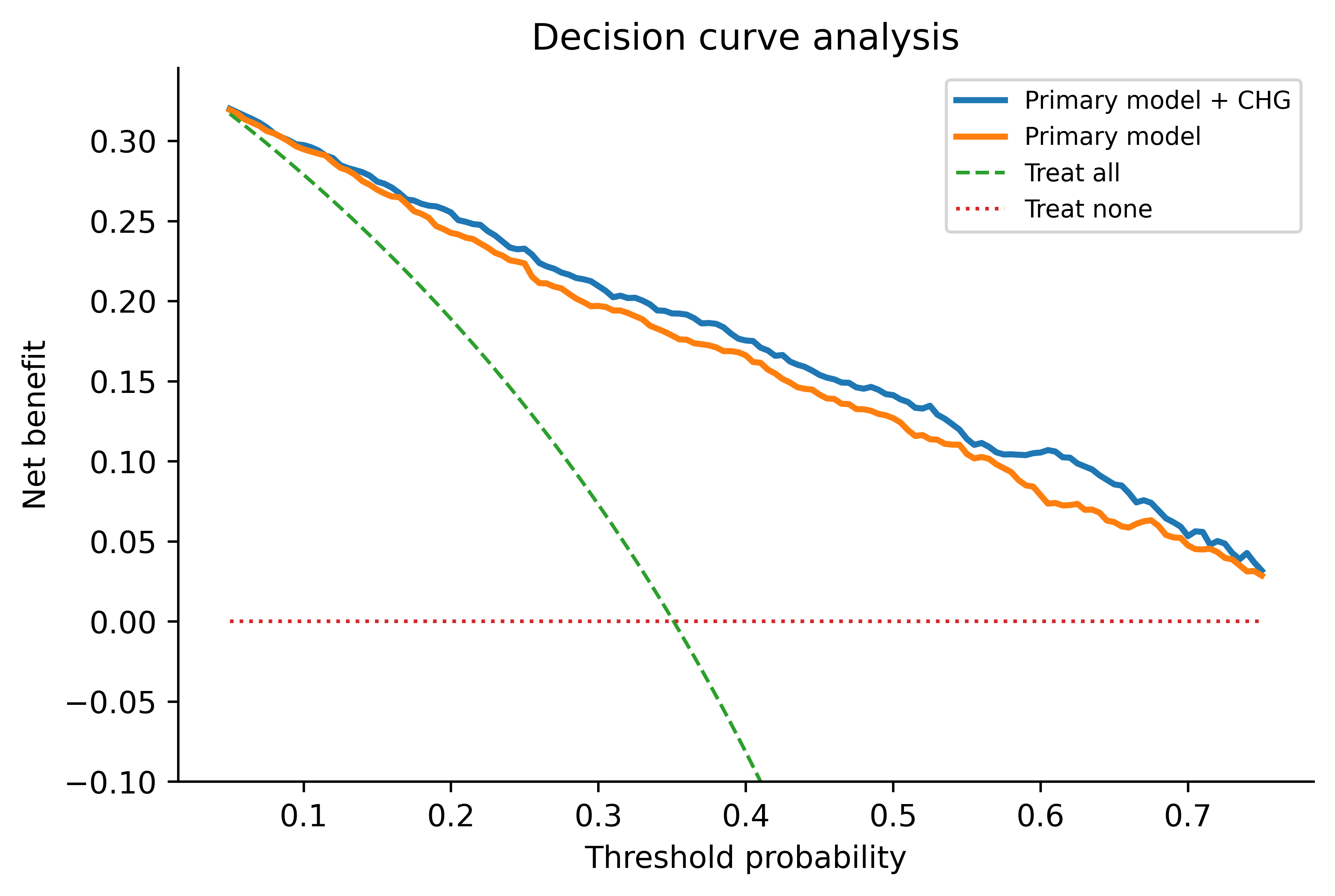


**Supplementary Figure S3.** Calibration plots for the primary clinical model and the primary clinical model plus CHG.


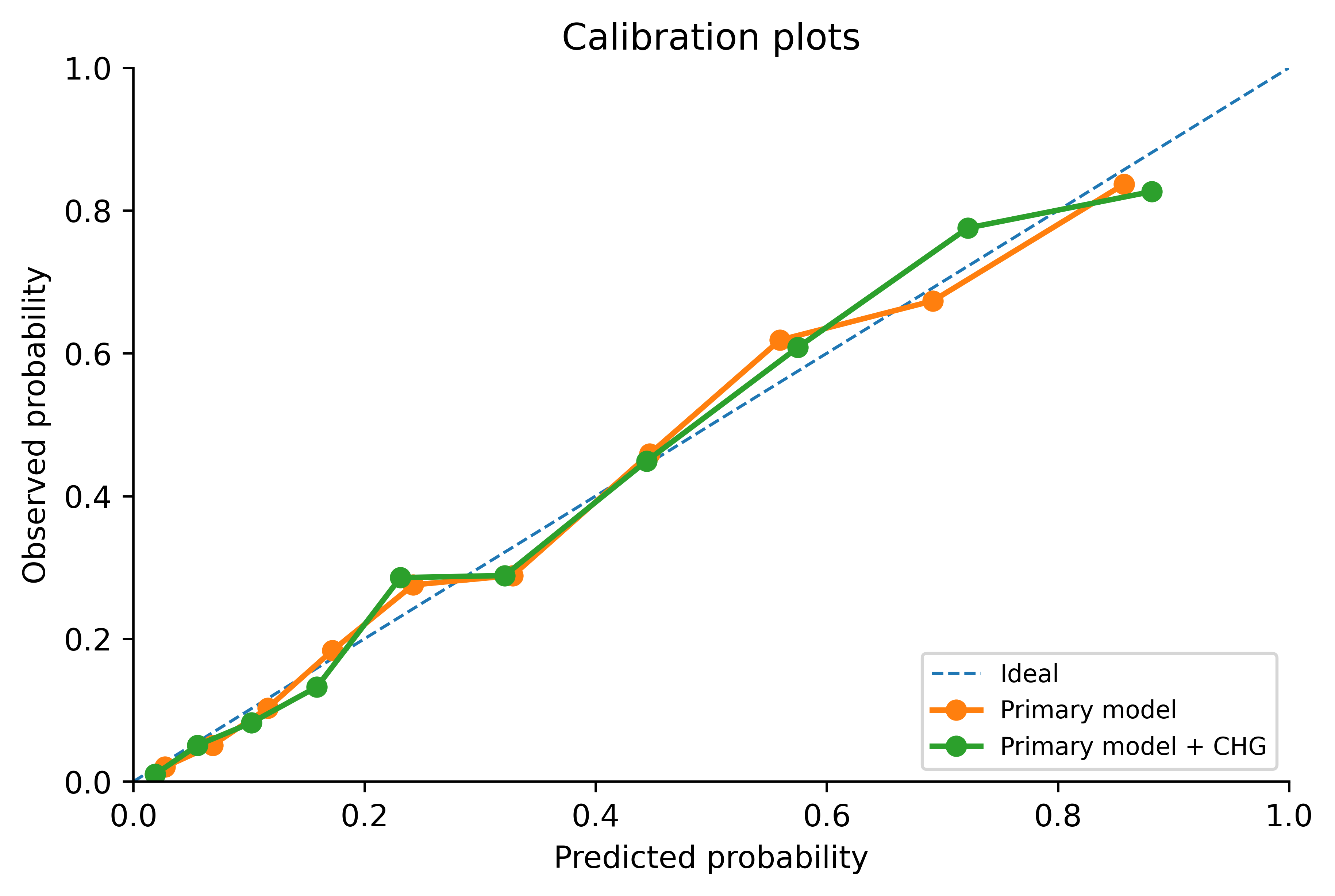

Supplement: Supplementary file 1 [file Supplementary_File_1.docx]
